# Fatty liver in men is associated with high serum levels of small, dense low-density lipoprotein cholesterol

**DOI:** 10.1186/1758-5996-4-34

**Published:** 2012-07-18

**Authors:** Kaori Hosoyamada, Hirofumi Uto, Yasushi Imamura, Yasunari Hiramine, Eriko Toyokura, Yoshihiro Hidaka, Tomomi Kuwahara, Ken Kusano, Kazuto Saito, Makoto Oketani, Akio Ido, Hirohito Tsubouchi

**Affiliations:** 1 Department of Internal Medicine, Kagoshima Kouseiren Hospital, 22-25 Tenpozan-cho, Kagoshima, 890-0061, Japan; 2 Digestive and Lifestyle Diseases, Kagoshima University Graduate School of Medical and Dental Sciences, 22-25 Tenpozan-cho, Kagoshima, 890-0061, Japan; 3 Digestive and Lifestyle Diseases, Kagoshima University Graduate School of Medical and Dental Sciences, 8-35-1 Sakuragaoka, Kagoshima, 890-8544, Japan; 4 Kagoshima Kouseiren Medical Health Care Center, 1-13-1 Yojiro, Kagoshima, 890-0062, Japan; 5 National Institute of Fitness and Sports in Kanoya, 1 Shiromizu-cho, Kanoya, 891-2393, Japan

**Keywords:** Small dense low-density lipoprotein, Fatty liver, Type 2 diabetes mellitus, Metabolic syndrome

## Abstract

**Aims:**

Our study addressed potential associations between fatty liver and small, dense low-density lipoprotein cholesterol (sd-LDL-C) levels using a cross-sectional analysis.

**Methods:**

We enrolled 476 male subjects. Serum sd-LDL-C concentrations were determined using precipitation assays.

**Results:**

Subjects were divided into four groups based on triglyceride (TG) and LDL-C levels: A, TG < 150 mg/dl and LDL-C < 140 mg/dl; B, TG < 150 mg/dl and LDL-C ≥ 140 mg/dl; C, TG ≥ 150 mg/dl and LDL-C < 140 mg/dl; and D, TG ≥ 150 mg/dl and LDL-C ≥ 140 mg/dl. sd-LDL-C levels and the prevalence of fatty liver were significantly higher in groups B, C, and D than in group A. Subjects were also categorized into four groups based on serum sd-LDL-C levels; the prevalence of fatty liver significantly increased with increasing sd-LDL-C levels. Additionally, logistic regression analysis revealed an independent association between sd-LDL-C concentrations and fatty liver using such potential confounders as obesity and hyperglycemia as variables independent of elevated TG or LDL-C levels.

**Conclusions:**

Fatty liver is a significant determinant of serum sd-LDL-C levels independent of the presence of obesity or hyperglycemia. Fatty liver may alter hepatic metabolism of TG and LDL-C, resulting in increased sd-LDL-C levels.

## Introduction

Atherogenic lipid profiles in patients with metabolic syndrome or glucose intolerance are characterized by hypertriglyceridemia, elevated apolipoprotein B levels, reduced high-density lipoprotein cholesterol (HDL-C) concentrations, and an increased proportion of small, dense low-density lipoprotein (sd-LDL) particles [1-3]. Compared with large LDL, sd-LDL particles show increased penetration of the arterial wall, lower affinity for the LDL receptor, longer half-life in plasma, greater susceptibility to glycation, and lower resistance to oxidative stress, suggesting that sd-LDL is highly atherogenic [4,5]. Indeed, patients with high levels of sd-LDL particles were shown to have an approximately 3-fold increase in the risk of developing coronary heart disease compared with individuals with primarily large, buoyant LDL particles [6]. In addition, the sd-LDL-C concentration has been suggested to be a better surrogate marker than the LDL-C concentration for the severity of coronary heart disease [7].

The presence of fatty liver is an independent predictor of coronary heart disease [8-10]. In addition, fatty liver is a manifestation of metabolic syndrome, and is associated with obesity, type 2 diabetes mellitus (T2DM), and hypertriglyceridemia [11]. In patients with T2DM or metabolic syndrome, fatty liver may enhance atherogenesis by increasing levels of sd-LDL particles [12,13]. The precise role of fatty liver in the pathogenesis of sd-LDL, however, is still unclear. In the present study, we performed a cross-sectional analysis of a cohort of 476 men to examine potential associations between the fatty liver and sd-LDL levels.

## Materials and methods

Four hundred seventy-six Japanese men who received regular health checkup in 2004 or 2005 participated in the study. Subjects were not taking medication for dyslipidemia and/or diabetes mellitus, and had serum triglyceride (TG) levels less than 400 mg/dl. This study was conducted at Kagoshima Kouseiren Medical Health Care Center, and was approved by the ethics committee of the Kagoshima Prefectural Federation of Agricultural Cooperatives for Health and Welfare.

Fatty liver diagnoses were made using ultrasonography (SSA-250A and SSA-700A, Toshiba, Ibaraki, Japan; Logic 400, GE Yokogawa, Tokyo, Japan) based on findings of a bright liver (increased echogenicity) with liver − kidney contrast (increased echogenicity of the liver compared with the right kidney). Body mass index (BMI) was calculated using the standard equation: body weight (kg)/height^2^ (m^2^). Obesity was defined as BMI values ≥ 25 kg/m^2^. Venous blood samples were obtained from all subjects before 9:00 am after an overnight fast and analyzed immediately. Alanine aminotransferase (ALT) and γ-glutamyl transpeptidase (γ-GTP) activities and serum concentrations of total cholesterol (TC), TG, and glucose were measured using standard laboratory procedures. HDL-C levels were determined using detergents and direct homogeneous assays of serum samples (Daiichi Chemicals, Takaoka, Japan). sd-LDL-C levels were determined using a previously described method with minor modifications [14,15] and a commercially available assay kit (sd-LDL SEIKEN, Denka Seiken Co., Tokyo, Japan). Subjects with TG levels ≥ 400 mg/dl were excluded from this study. LDL-C concentrations were calculated using the Friedewald formula: LDL-C (mg/dl) = TC (mg/dl) − HDL-C (mg/dl) − TG (mg/dl)/5. Serological testing for hepatitis B surface antigen (HBs Ag) and hepatitis C virus antibodies (HCV Ab) was performed using an enzyme immunoassay and enzyme-linked immunosorbent assay, respectively.

Patients were defined as hypertension based on systolic blood pressure ≥ 130 mmHg, diastolic blood pressure ≥ 85 mmHg, or if they were taking medication for hypertension. Hyperglycemia or diabetes was identified based on fasting blood glucose levels ≥ 110 mg/dl or ≥ 126 mg/dl, respectively. Hypertriglyceridemia and elevated levels of LDL-C were defined as TG levels ≥ 150 mg/dl and LDL-C levels ≥ 140 mg/dl, respectively [16]. A common questionnaire was used by a public health nurse to assess each subject’s history of alcohol intake and smoking status. Current alcohol consumption was defined as daily alcohol intake of ≥ 20 g/day.

### Statistical analysis

Continuous variables were analyzed using *t*-tests or analysis of variance (ANOVA), and categorical variables were examined using chi-square tests. Multiple comparisons among the differences were examined using Dunnett’s or Ryan’s method. Maximum likelihood of odds ratios for fatty liver risk and 95% confidence intervals (95% CIs) were calculated using logistic regression models. All presented *P* values are two-sided. *P* values < 0.05 were considered significant. Statistical analyses were performed using JMP version 7 (SAS Corp, Cary, NC, USA) and R version 2.13.0.

## Results

### Characteristics of the subjects, clinical parameters, and relationships to the presence of fatty liver

Subject demographics, clinical parameters, and associations with fatty liver are summarized in Table 1. Among 476 subjects, 190 (39.9%) were diagnosed with fatty liver (Table 1). The mean concentration [95% CI] of sd-LDL-C among all subjects was 44.6 mg/dl [43.1 − 46.2]. The prevalences of hyperglycemia and diabetes were 29.0% and 8.0%, respectively.

**Table 1:** Characteristics of the study subjects

	**Overall**	**Fatty liver (−)**	**Fatty liver (+)**	***P*values**
	**(476)**	**(286)**	**(190)**	**Fatty liver (−) vs.(+)**
Age (years)	55.9 [55.1−56.8]	57.8 [56.5−58.9]	53.2 [51.9−54.4]	< 0.001
BMI (kg/m^2^)	24.2 [23.9−24.5]	23.1 [22.7−23.4]	25.9 [25.5−26.3]	< 0.001
Obesity (% of subjects)	37.0	23.8	56.8	< 0.001
Blood glucose (g/dl)	106.8 [105.2−108.4]	104.5 [102.4−106.5]	110.2 [107.7−112.8]	< 0.001
Hyperglycemia (% of subjects)	29.0	24.5	35.8	0.008
TG (mg/dl)	132.5 [125.9−139.2]	112.6 [104.5−120.7]	162.6 [152.7−172.5]	<0.001
HDL-C (mg/dl)	55.7 [54.5−56.9]	58.0 [56.4−59.6]	52.2 [50.5−53.9]	< 0.001
LDL-C^$^ (mg/dl)	130.2 [127.6−132.9]	127.4 [124.1−130.7]	134.5 [130.1−138.8]	0.010
TC (mg/dl)	212.4 [209.5−215.3]	208.0 [204.3−211.7]	219.1 [214.4−223.9]	< 0.001
sd-LDL-C (mg/dl)	44.6 [43.1−46.2]	40.8 [38.9-42.8]	50.5 [48.0-53.0]	< 0.001
Apolipoprotein B (mg/dl)	97.8 [96.−99.6]	93.2 [91.0−95.4]	104.6 [101.9−107.3]	< 0.001
ALT (IU/L)	28.1 [26.4−29.7]	22.7 [20.8−24.6]	36.1 [33.7−38.5]	< 0.001
γ-GTP (IU/L)	45.4 [41.2−49.5]	36.9 [31.7−42.1]	58.0 [51.6−64.4]	< 0.001
Hypertension (% of subjects)	54.8	52.5	58.4	0.200
Current alcohol consumption (%)	37.8	40.2	34.2	0.186
Smoking status (% of subjects) (never/former/current)	31.5/39.9/28.6	31.8/39.5/28.7	31.1/40.5/28.4	0.974

Data are expressed as means [95% CIs] or percentages.

^$^, LDL-C levels were calculated using the Friedwald formula.

Continuous variables were compared by *t*-test and categorical variables were compared by Chi-square test.

Of note, the lipid profiles of subjects with fatty liver were typically atherogenic (Table 1); the concentrations of TC, TG, LDL-C, sd-LDL-C, and apolipoprotein B were significantly higher in the group with fatty liver than in the subjects without fatty liver. In addition, the concentration of HDL-C was significantly lower in the fatty liver group than in the subjects without fatty liver.

Subjects with fatty liver were significantly younger than those without fatty liver (Table 1). BMI values, the prevalence of obesity, serum glucose concentrations, and the prevalence of hyperglycemia were significantly higher in the fatty liver group compared to the subjects without fatty liver. No significant differences were noted between the groups in the prevalence of hypertension, current alcohol consumption, and smoking status.

### Contributions of hypertriglyceridemia and elevated levels of LDL-C to serum sd-LDL-C concentrations and the prevalence of fatty liver

Features of four groups categorized based on the presence of elevated LDL-C levels (≥ 140 mg/dl) and/or hypertriglyceridemia (≥ 150 mg/dl) are shown in Table 2.sd-LDL-C concentrations were higher in the group with only elevated levels of LDL-C (group B; *P* < 0.001) and the group with only hypertriglyceridemia (group C; *P* < 0.001) compared with the control group (group A). Additionally, sd-LDL-C concentrations in the group with elevated levels of LDL-C and hypertriglyceridemia were the highest among the groups (Group D; *P* < 0.001 compared with the control group). Compared with the control group, the groups with hypertriglyceridemia had higher proportions of sd-LDL-C (group C and group D; both *P* < 0.001), whereas group B did not show a significant difference (*P* = 0.766).

**Table 2:** Comparison of groups categorized based on the presence of elevated LDL-C levels and hypertriglyceridemia

**Elevated LDL-C levels/hypertriglyceridemia**	**Group A**	**Group B**	**Group C**	**Group D**	***P*value**
	**(−)/(−) (n = 216)**	**(+)/(−) (n = 120)**	**(−)/(+) (n = 95)**	**(+)/(+) (n = 45)**	
Lipid profiles					
TG (mg/dl)	91.1 [85.1−96.7]	98.1 [90.5−105.6]	229.3 [220.8−237.8]	219.0 [206.7−231.4]	< 0.001
HDL-C (mg/dl)	59.3 [57.6−61.1]	56.8 [54.4−59.1]	49.3 [46.7−52.0]	48.9 [45.1−52.7]	< 0.001
LDL-C (mg/dl)	114.2 [111.8−116.7]	162.5 [159.2−165.8]	112.1 [108.4−115.8]	159.1 [153.8−164.5]	< 0.001
Sd-LDL-C (mg/dl)	33.8 [31.9−35.6]	49.6 [47.2−52.1]	51.9 [49.2−54.7]	68.5 [64.5−72.5]	< 0.001
Sd-LDL-C/LDL-C	0.30 [0.28−0.31]	0.30 [0.29−0.32]	0.47 [0.45−0.48]	0.43 [0.40−0.46]	< 0.001
TC (mg/dl)	191.8 [188.7−194.8]	238.9 [234.8−243.1]	207.3 [202.7−212.0]	251.8 [245.1−258.6]	< 0.001
Apolipoprotein B (mg/dl)	82.8 [75.5−80.4]	112.6 [110.3−114.9]	99.4 [96.8−102.0]	126.3 [122.5−130.1]	< 0.001
Demographics, clinical characteristics, and laboratory data					
Age (years)	57.4 [56.2−58.7]	56.1 [54.4−57.8]	54.3 [52.4−56.3]	51.5 [48.7−54.3]	< 0.001
BMI (kg/m^2^)	23.6 [23.2−24.0]	24.0 [23.4−24.5]	25.1 [24.5−25.7]	25.7 [24.8−26.6]	< 0.001
Obesity (% of subjects)	31.0	30.8	46.3	62.2	< 0.001
Glucose (mg/dl)	103.8 [101.4−106.1]	105.2 [102.1−108.4]	113.5 [110.0−117.1]	111.5 [106.4−116.7]	< 0.001
Hyperglycemia					
(% of subjects)	22.7	27.5	37.9	44.4	0.004
ALT (IU/L)	24.3 [22.0−26.6]	27.4 [24.3−30.5]	34.4 [30.9−37.9]	34.6 [29.5−39.6]	< 0.001
γ-GTP (IU/L)	35.3 [29.4−41.2]	38.6 [30.7−46.5]	70.0 [61.2−78.9]	59.6 [46.8−72.5]	< 0.001
Fatty liver (% of subjects)	25.9	43.3	61.1	53.3	< 0.001
Hypertension					
(% of subjects)	53.7	54.2	57.9	55.6	0.919
Current alcohol consumption					
(% of subjects)	38.4	54.2	46.3	33.3	0.153
Current smokers					
(% of subjects)	28.7	30.0	28.4	24.4	0.919

Data are expressed as means [95% CIs] or percentages. ^$^, LDL-C levels were calculated using the Friedwald formula.

Continuous variables were compared by ANOVA and categorical variables were compared by Chi-square tests.

No significant differences were observed between groups A and B in BMI values (*P* = 0.719), the prevalence of obesity, serum glucose levels (*P* = 0.838), and the prevalence of impaired fasting glucose levels. The prevalence of fatty liver in group B (*P* = 0.001), however, was significantly higher than in group A. No significant difference between groups A and B was noted for the concentration of HDL-C (*P* = 0.235). BMI values were significantly higher in group C than in group A (*P* = 0.001). The prevalence of obesity was higher in group C than in group A, although the difference was not significant (Ryan’s method).

The mean ages (*P* = 0.024), blood glucose levels (*P* = 0.021), and rates of hyperglycemia (*P* = 0.006) significantly differed in groups A and C. Furthermore, the prevalence of fatty liver was significantly higher in group C than in group A (*P* < 0.001). The concetration of HDL-C was lower in group C than in group A (*P* < 0.001).

Compared with group A, group D had higher BMI values (*P* < 0.001), blood glucose concentrations (*P* < 0.001), and rates of obesity (*P* = 0.003), hyperglycemia (*P* = 0.003), and fatty liver (*P* < 0.001), whereas the mean age (*P* < 0.001) and HDL-C concentration (*P* < 0.001) were lower.

### Independent association between fatty liver and sd-LDL-C levels

The subjects were classified into four groups based on serum sd-LDL-C concentrations: ≤ 21.1 mg/dl, 21.2–42.7 mg/dl, 42.8–55.7 mg/dl, and ≥ 55.8 mg/dl. Characteristics of the groups are summarized in Table 3. Among these groups, the concentrations of TG, LDL-C, TC, and apolipoprotein B increased significantly and the HDL-C concentration decreased significantly as sd-LDL-C levels increased. BMI values and the rates of fatty liver and hyperglycemia increased significantly with increasing sd-LDL-C levels. No significant association was detected between sd-LDL-C levels and the prevalence of hypertension, current alcohol consumption, or current smoking.

**Table 3:** Characteristics of groups categorized based on serum sd-LDL-C concentrations

**Characteristics**	**Quartiles of serum sd-LDL-C concentrations (mg/dl)**				
	**≤ 21.1 (n = 119)**	**21.2 - 42.7 (n = 119)**	**42.8 - 55.7 (n = 118)**	**55.8 (n = 120)**	***P*value**
Lipid profiles					
TG (mg/dl)	83.8 [72.6−95.1]	111.8 [100.5−123.1]	145.3 [134.0−156.7]	188.7 [177.5−200.0]	< 0.001
HDL-C (mg/dl)	63.5 [61.2−65.8]	57.6 [55.3−59.8]	52.7 [50.4−55.0]	49.0 [46.7−51.3]	< 0.001
LDL-C^$^ (mg/dl)	110.3 [105.7−115.0]	125.9 [121.2−130.6]	135.9 [131.2−140.5]	148.6 [144.0−153.3]	< 0.001
TC (mg/dl)	190.6 [185.6−195.7]	205.8 [200.8−210.9]	217.7 [212.6−222.8]	235.4 [230.4−240.4]	< 0.001
Apolipoprotein B (mg/dl)	78.0 [75.5−80.4]	91.9 [89.4−94.4]	103.5 [101.0−105.9]	117.6 [115.1−120]	< 0.001
Demographics, clinical characteristics, and laboratory data					
Age (years)	57.9 [56.1−59.6]	56.8 [50.1−58.6]	55.3 [53.6−57.1]	53.8 [52.0−55.5]	0.007
BMI (kg/m^2^)	23.4 [22.8−23.9]	24.0 [23.4−24.5]	24.5 [24.0−25.1]	25.0 [24.4−25.5]	< 0.001
Obesity (% of subjects)	30.3	30.3	39.8	47.5	0.017
Glucose (mg/dl)	105.0 [101.8−108.2]	105.6 [103.0−106.2]	10.6.2 [103.8−108.6]	110.4 [105.9−115.0]	0.080
Hyperglycemia					
(% of subjects)	20.2	28.6	30.5	36.7	0.045
ALT (IU/L)	22.4 [19.3−25.5]	26.5 [23.3−29.6]	29.1 [26.0−32.3]	34.3 [31.2−37.4]	< 0.001
γ-GTP (IU/L)	31.9 [23.8−39.9]	40.5 [32.4−48.6]	47.0 [38.9−55.1]	62.0 [53.9−70.0]	< 0.001
Fatty liver (% of subjects)	23.5	34.5	44.0	57.5	< 0.001
Hypertension					
(% of subjects)	52.2	52.1	53.4	61.7	0.380
Current drinking					
(% of subjects)	39.5	39.5	39.8	32.5	0.587
Current smoking					
(% of subjects)	23.5	32.8	25.4	32.5	0.262

Data are expressed as means [95% corresponding intervals] or percentages. ^$^, LDL-C levels were calculated using the Friedwald formula.

Continuous variables were compared by ANOVA and categorical variables were compared by Chi-square tests.

As shown in Table 4, logistic regression analysis revealed an independent association between sd-LDL-C levels and the presence of fatty liver using BMI values, the presence of hyperglycemia, and other potential confounders as variables. This association remained when the analysis was performed only with subjects who did not have elevated levels of LDL-C (subjects in groups A and C from Table 2) or with those who did not have hypertriglyceridemia (subjects in groups A and B from Table 2).

**Table 4:** Association between fatty liver and sd-LDL-C levels

	**Quartiles of serum sd-LDL-C concentrations (mg/dl)**				
**Characteristics**	**≤ 21.1**	**21.2 - 42.7**	**42.8 - 55.7**	**≥ 55.8**	***P*value for trend**
All subjects	1 (referent)	1.59 [0.85−3.20]	1.96 [1.05−3.69]	2.48 [1.32−4.70]	0.001***
Subsets of subjects					
HBs-Ag (−), HCV-Ab(−)^#^	1 (referent)	1.83 [0.85−4.05]	2.50 [1.18−5.43]	3.60 [1.73−7.77]	0.001***
Elevated LDL-C levels (−) ^##^	1 (referent)	1.60 [0.79−3.26]	1.95 [0.92−4.16]	2.89 [1.29−6.62]	0.009***
Hypertriglyceridemia (−) ^###^	1 (referent)	1.14 [0.57−2.29]	1.55 [0.76−3.23]	2.25 [1.00−5.10]	0.018***

Risk of fatty liver was estimated by logistic regression analysis using sd-LDL-C levels, age, BMI, hypertension, hyperglycemia, current alcohol consumption, and current smoking as covariables. Data are expressed as odds ratios [95% corresponding intervals]. *P* values for trend were obtained using the likelihood ratio test. ^#^, Subjects were 385 men who were negative for both HBs-Ag and HCV-Ab. ^##^, Subjects were 311 men who did not have elevated levels of LDL-C. ^###^, Subjects were 336 men who did not show hypertriglyceridemia.

## Discussion

This study provides important information about the relationship between fatty liver and LDL particle size. We revealed an independent association between the presence of fatty liver and serum sd-LDL-C levels, including after we adjusted for such potential confounders as BMI and impaired fasting glucose levels. It may be problematic to discuss visceral obesity and insulin resistance only based on BMI and impaired fasting glucose levels, respectively. Toledo and colleagues, however, showed a relationship between fatty liver and sd-LDL size in patients with T2DM [12]. Sugeno and colleagues suggested that fatty liver synergistically interacts with metabolic syndrome to affect sd-LDL-C levels [13]. Based on these studies and the data present here, fatty liver appears to affect LDL particle size, an effect that may be independent of visceral obesity and systemic insulin resistance. Thus, treating fatty liver may decrease atherogenesis in the patients with metabolic syndrome or T2DM by reducing sd-LDL-C levels.

The composition of TG and cholesterol esters in LDL particles is modified through interactions with TG-rich lipoproteins and cholesterol ester transfer protein (CETP); the molecules are sequentially hydrolyzed by lipoprotein lipase, resulting in the generation of sd-LDL particles [1]. Therefore, elevated LDL-C levels and hypertriglyceridemia seem to be the causative dyslipidemia of sd-LDL particles formation. Tokuno and colleagues reported that statin and fibrate decrease sd-LDL-C concentrations in patients with T2DM via different mechanisms: the former does not affect the sd-LDL-C/LDL-C ratio, whereas the latter reduces this ratio [17]. In the present study, sd-LDL-C concentrations were significantly elevated in subjects with elevated LDL-C levels and normal TG levels, although the sd-LDL-C/LDL-C ratio did not increase. In contrast, sd-LDL-C concentrations and the sd-LDL-C/LDL-C ratio were elevated in subjects with normal LDL-C levels and hypertriglyceridemia (Table 2). Notably, fatty liver was significantly more common in both of these groups. In addition, as shown in Table 4, an independent association between fatty liver and sd-LDL-C levels was observed when multivariable analysis was performed using only subjects without elevated LDL-C levels (subjects in groups A and C from Table 2) or those without hypertriglyceridemia (subjects in groups A and B from Table 2). Therefore, fatty liver appears to independently affect LDL particle size owing, at least in part, to impaired hepatic metabolism of TG and LDL-C. The concentration of sd-LDL-C were highest in the group with elevated LDL-C levels and hypertriglyceridemia (group D from Table 2), suggesting that the effects of impaired metabolism of TG and LDL-C may be additive.

Visceral obesity and insulin resistance have been recognized as major causes of increased levels of sd-LDL particles, because these factors are major contributors to postprandial hypertriglyceridemia; one underlying mechanism is increased free fatty acid release from adipocytes, which stimulates hepatic TG output. Additionally, if a fatty liver is present, upregulated de novo synthesis of fatty acids may increase hepatic TG production. Donnely et al. reported that approximately 60% of fat that accumulates in the liver and is incorporated into lipoprotein is derived from circulating free fatty acids, and nearly 25% results from de novo lipid synthesis in patients with nonalcoholic fatty liver disease (NAFLD) [18]. In addition to altered TG output, fatty liver has been shown to be associated with increased TG content per very-low-density lipoproteins (VLDL) particle which is defined as large VLDL [12,19]. Large VLDL efficiently promotes modification of LDL particles via CETP. Recent studies revealed that the liver X receptor (LXR)-sterol regulatory element-binding protein (SREBP)-1c pathway governs the size of VLDL particles secreted by the liver [20,21]. It is noteworthy that the LXR-SREBP-1c pathway is a major causative factor of fatty liver, because several genes involved in de novo fatty acid synthesis are expressed in response to upregulated LXR-SREBP-1c signaling [22]. Thus, fatty liver affects VLDL particles quantitatively and qualitatively, resulting in increased sd-LDL formation.

Interestingly, the LXR-SREBP-1c pathway is a key regulator of not only fatty acid metabolism but also cholesterol metabolism [23,24]. Activation of the LXR-SREBP-1c pathway by increased intrahepatic cholesterol levels stimulates cholesterol secretion in VLDL and suppression of LDL uptake. Enhanced de novo synthesis of cholesterol in livers of patients with NAFLD [25] may increase intrahepatic cholesterol concentrations. Excess intake of dietary cholesterol has been observed in patients with NAFLD [26]. We previously showed a positive independent association between the presence of fatty liver and serum cholesterol levels in men [27]. Thus, we believe that fatty liver is independently associated with elevated serum LDL-C levels owing to altered cholesterol metabolism that results in increased sd-LDL-C levels.

The activities of CETP and hepatic lipase may correlate with sd-LDL-C levels [28]. Lipoprotein lipase is responsible for an important step in TG clearance. Prolonged accumulation of TG-rich remnants following meals may also be associated with postprandial dyslipidemia. Thus, further studies are needed to clarify the role of fatty liver in elevated levels of sd-LDL particles. Of note, our study has several limitations. First, the association between fatty liver and sd-LDL-C was examined with multivariate analysis using age, BMI, hypertension, hyperglycemia, current alcohol consumption [29], smoking status [30], and sd-LDL-C levels as covariables. It is possible, however, that additional factors that we did not analyze may have affected the results. For example, we did not enroll women subjects, which may have skewed the results [30]. Second, the diagnosis of fatty liver was made using abdominal ultrasonography, which identifies fatty steatosis. Ultrasonography may not detect a subset of advanced alcohol or nonalcoholic fatty liver diseases—referred to as burnt-out steatohepatitis—which are characterized by less fatty steatosis.

## Conclusion

Fatty liver may affect the hepatic metabolism of TG and/or LDL-C, resulting in increased serum sd-LDL-C levels and accelerated atherogenesis in patients with metabolic syndrome or T2DM. Therefore, fatty liver should be treated, especially if patients present with metabolic syndrome or T2DM. Further studies, however, are needed to develop effective treatment strategies for fatty liver.

## Abbreviations

LDL-C: Low-density lipoprotein cholesterol; sd-LDL-C: Small, dense low-density lipoprotein cholesterol; TG: Triglyceride; T2DM: Type 2 diabetes mellitus; HDL-C: High-density lipoprotein cholesterol; BMI: Body mass index; ALT: Alanine aminotransferase; γ-GTP: γ-glutamyl transpeptidase; TC: Total cholesterol; NAFLD: Nonalcoholic fatty liver disease; VLDL: Very-low-density lipoproteins.

## Competing interests

All authors declare that they have no conflict of interest.

## Authors’ contributions

KH, HU and YI researched and analyzed data. HT also participated in the concept and design of the study, interpretation of data and reviewed/edited the manuscript. YH, ET, YH and TK collected the data. KS analyzed data. MO and AI contributed to discussion and wrote the manuscript. All authors read and approved the final version of the manuscript.
